# Gender differentials in cricket farming and its impact on household food security levels in East Africa

**DOI:** 10.1371/journal.pone.0326108

**Published:** 2025-06-25

**Authors:** Nancy Ndung’u, Hezron Isaboke, Wilckyster Nyarindo, Mark Otieno, Mathew Gicheha, John Kinyuru

**Affiliations:** 1 Department of Agricultural Economics and Extension, University of Embu, Embu, Kenya; 2 Department of Water and Agricultural Resource Management, University of Embu, Embu, Kenya; 3 Department of Animal Science, Jomo Kenyatta University of Agriculture and Technology, Nairobi, Kenya; 4 Department of Food Science and Technology, Jomo Kenyatta University of Agriculture and Technology, Nairobi, Kenya; Lusofona University of Humanities and Technologies: Universidade Lusofona de Humanidades e Tecnologias, PORTUGAL

## Abstract

Crickets, as one of the edible insects, represent a promising alternative for enhancing food security through direct human consumption or as livestock feed. This study investigated the impact of cricket farming on household food security in Kenya and Uganda, focusing on a sample of 187 cricket farmers and 457 non-farmers. Utilizing the Food Insecurity Experience Scale and the Food Consumption Score, the research assesses dimensions of food access, stability, utilization, and availability. An endogenous switching regression model was employed to analyze the influence of gender of cricket farming decision-makers and participation in cricket farming on food security outcomes. For male decision-makers, key determinants for adoption included awareness, the availability of processing technology, ready markets, and perceived risks. Female decision-makers were more significantly influenced by awareness, training opportunities, perceived benefits, and social norms. Female decision-makers who engaged in cricket farming (Y1 = 1.756) exhibited a higher levels of household food security compared to non-adopters (Y0 = 1.567), yielding a significant positive treatment effect (ATT = 0.188). In contrast, male adopters experienced a slight decrease in food security, reflecting a negative effect (ATT = −0.516). This study highlights the importance of gender differences in food security outcomes and informs policy initiatives aimed at promoting sustainable food security through cricket farming. Targeted training is recommended to enhance female farmers’ skills and improve productivity as a proxy for food security. Interventions should promote group association and establish aggregation centers at the policy level to enhance access to resources and market linkages for male farmers.

## 1. Introduction

Food and nutrition security is a global concern linked to the Sustainable Development Goals (SDGs), specifically SDG 2 [1]. Despite the significance of food and nutrition security, the global assessment of hunger reveals a lack of progress in achieving the Zero Hunger goal, particularly in the African region [2]. Women are central in household food security as food producers, consumers, homemakers, and agents for social change [3,4]. In sub-Saharan Africa, women account for 70–80% of individuals in sustainable food production [5,6]. However, they face obstacles such as limited access to information and resources, unfavorable policies, lack of education, and social and cultural norms [7]. Additionally, individuals and communities historically marginalized, such as women and small-scale farming households, often struggle to influence food security and the broader food system, as indicated in the High-Level Panel of Experts (HLPE) report of 2020. Consequently, these groups are more vulnerable to food insecurity. By supporting women in agriculture, the region can achieve more resilient and productive food systems [8].

The presence of more than 470 species of edible insects in Africa [9,10], along with their potential as an alternative to conventional animal proteins [10,11] presents a promising solution to the challenges of food and nutrition insecurity, thereby contributing to the achievement of SDG 2. Edible insects are highly nutritious making them a valuable food source, particularly for low-income households. They contain sufficient levels of proteins, fats and micronutrients for human nutrition [12,13] and a high protein source for animal feed [14]. These insects have gained recognition for their efficient feed conversion, which requires less land and water and emits fewer greenhouse gases compared to conventional protein sources [15–17]. In addition to their environmental benefits, insect farming offers opportunities for job creation, economic growth, and improved food security [18,19]. Edible insects are traditionally consumed in East Africa (EA) [20,21], and several studies have shown a resurgence in consumer acceptance [22–25]. Initiatives by non-governmental organizations (NGOs), development agencies, public–private partnerships, and supportive policy frameworks in East AFrica have sought to promote cricket farming for human consumption [14,26,27], with farming/production systems piloted and scaled in Kenya and Uganda. In EA, efforts have been undertaken to increase acceptance in consumption of insects, crickets in particular [28–31], and the implementation of legislation [32,33] which highlight the growing recognition of edible insects as a viable food security and sustainability solution. Despite these efforts, this value chain in East Africa remains underdeveloped, characterized by low farmer participation [34], small-scale production systems [26,35] and a limited range of value-added products [36]. As a result, crickets and cricket-based products are rarely available in mainstream markets, and the transition from small scale farming to large-scale commercialization remains challenging [37,38]. This raises questions about how the participation of male and female farmers in cricket production might affect household food security.

In African context, women have played a leading role in the edible insect value chain, actively participating in the collection, processing, and preparation of insects for food [39–41]. Recent study revealed that women constitute over two-thirds of participants in cricket farming in East Africa, highlighting their active role in this emerging sector and its potential for economic empowerment [37]. However, there is a lack of comprehensive data regarding the extent of smallholders’ involvement in insect farming and its implications for household food security in East Africa. This study addressed a significant gap in the literature by examining not only the role of insect farming as a food source but also its viability as an economic activity in East Africa. While prior research has emphasized the potential of insect farming as a sustainable source of nutrition [18,19,38,42,43], efforts toward commercialization have encountered challenges related to supply limitations, including restricted farmer participation and small production scales, which hinder market expansion. This research specifically tackled these gaps by investigating the supply-side dynamics of cricket farming and its integration into broader household strategies aimed at improving food security. To capture the nuanced dynamics of adoption and their implications for food security, the study utilized detailed survey data in conjunction with robust econometric models, such as multinomial regression and endogenous switching regression. Hence, elucidating how smallholder involvement in cricket farming intersects with gender dynamics and socio-economic factors, providing new insights into promoting scalable and sustainable cricket farming that enhances both nutritional outcomes and economic opportunities.

In the field of gender studies, the gender of the household head has predominantly been employed as a determinant of gender differences in agricultural technologies. Kassie et al [44], established that food security of female-headed households (FHHs) could significantly improve if they possessed levels of resource utilization comparable to those of male-headed households (MHHs). Matere et al. [45] findings indicated that, while overall adoption resulted in increased yields, MHHs benefited more substantially due to superior access to resources. In contrast, FHHs faced barriers such as limited access to inputs and extension services. The reliance on the gender of the household head as a measure of gender participation in agriculture is fundamentally flawed, as it fails to recognize the contributions of women who are actively engaged in agricultural decision-making within male-headed households. Numerous studies utilize household head gender as a proxy for decision-making authority; however, this approach inadequately captures the complexities of intra-household dynamics, wherein women may assume the role of primary decision-makers for specific activities [46]. A more nuanced analytical approach is necessary to acknowledge women’s roles as decision makers within agricultural enterprises [47]. In this study, the gender to participation in cricket farming is based on the decision-maker on adoption rather than the gender of the household head.

Agricultural innovation adoption plays a pivotal role in enhancing household food security (HHFS), with advancements such as improved maize varieties, fertilizers, and irrigation technologies demonstrating a positive impact [48–51]. This study expands the existing research by exploring cricket farming as an innovative agricultural alternative and its potential effects on household food security (HHFS). The choice to investigate cricket farming is driven by gaps in prior research, which has mainly concentrated on conventional protein sources while overlooking alternative protein sources, such as edible insects. Gender emerges as a critical determinant of food security outcomes; a consideration that directly informs our research hypothesis on differential adoption rates Prior studies indicate that female farmers are often early adopters of agricultural innovations and prioritize household food security [3,52–54] which justifies the inclusion of gender as a key variable in the study. Furthermore, male-headed households (MHHs) experience higher yields as a proxy for food security in comparison to female-headed households (FHHs) [45,55]. Thus, empowering women has been shown to positively influence household food security and dietary outcomes [51,55,56]. Cricket farming serves as a potential equalizing factor in food production by offering a low-cost, accessible alternative to improve household nutrition and enhance economic resilience.

The role of supportive policies, association membership and institutional frameworks in facilitating technology adoption [48,52,53,57,58], has also guided this study. The provision of research and extension services is also a key factor in facilitating the adoption of technologies that improve food security outcome [48–50]. This study included association membership and availability of training in handling EIs variables because existing evidence suggests that these factors improve technology uptake. The study assessment also incorporated household characteristics, including household size and farming experience [51,58,59], which are recognized as determinants of food security. Land size, under specific agricultural activities, has been shown to positively influence food security by increasing annual food expenditure per capita [53]. Our study examines how cricket farming, which requires minimal land, survive harsh climatic conditions [60], provides an alternative for smallholder farmers with limited land resources. While income [57,61], is a key indicator of food security, research suggests that assets may serve as a stronger predictor. Studies have produced mixed findings regarding the impact of income on adoption of cricket farming; for instance, Musungu et al. [38] reported an increase in participation associated with economic opportunities, whereas Halloran et al. [62] documented a decline. While income can enhance food security in certain contexts [58,61], this inconsistency highlights the need to incorporate asset-based measures in food security assessments, particularly in emerging economies where structural barriers and socio-economic constraints influence food security outcomes. Asset ownership is a stronger predictor as it facilitates production, provides a buffer against climate shocks, and ensures long-term resilience [63–65]. Therefore, this study prioritized assets-based variables to provide a more comprehensive understanding of food security within cricket-farming households.

Beyond economic considerations, unobserved factors such as social norms, risk aversion, and psychological elements influence food security outcomes [45] especially for novel foods such as crickets. Participation in food security interventions has been associated with improved food security outcomes, including increased per capita calorie intake [56,66]. Given this context, this study examined both the economic factors and the social and behavioral aspects that affect the adoption of cricket farming.

Thus, the study investigated the impact of edible insect farming on household food security. Analysing the benefits of cricket farming for households in Kenya and Uganda provides valuable insights for shaping policies and interventions to promote it as a sustainable solution to food insecurity. The primary objective is to compare the effect of male and female farmer participation in cricket farming on household food security levels in Kenya and Uganda. The paper examined how cricket farming influenced household food security across gender lines among smallholders. The central research question is: “How does engagement in cricket farming by male and female farmers influence food security in households in Kenya and Uganda?” Additionally, this research aims to deepen understanding of how edible insects contribute to the eradication of hunger and malnutrition.

## 2. Theoretical frameworks

Household Utility Maximization Theory (HUMT) posits that households, functioning as rational economic entities, make decisions aimed at maximizing their utility (satisfaction) given their available resources, constraints and expected benefits [67]. This framework facilitated the comparison of scenarios in which a household is faced with the decision to engage in the farming of crickets or to abstain from such activities, ultimately opting for the alternative that provides the highest utility [67]. In the context of cricket farming, gender dynamics could affect labor allocation, control over income, and the adoption of farming technologies. Additionally, awareness is associated with creating information on benefits and risks associated with farming, hence the decision maker will weigh the potential utility against risks to ascertain if cricket farming supports their food security strategy

Furthermore, the study employed a related Theory of Reasoned Action (RAA), to elucidate that household decisions are also swayed by knowledge and perception of the technology. The RAA suggests that an individual’s likelihood of engaging in a specific behavior is shaped by their attitude, perceived social norms, and the necessary abilities and skills (behavioral control), which may either facilitate or hinder their participation [68]. In the context of adopting cricket farming, awareness shape attitudes and subjective norms, where attitudes are more favorable when households are informed about nutritional and economic advantages. Perceived benefits align with the attitude component of RAA, while perceived norms align with social norms whether cricket farming and consumption is socially acceptable. Perceived risks further impact the behavioral control aspects of the RAA, should a household perceive cricket farming as risky, they may hesitate to adopt it.

## 3. Materials and methods

### 3.1. Study area and data source

We surveyed farming households in Siaya and Kisumu counties (Kenya) and Masaka District (Uganda) using purposive sampling to identify cricket producers, followed by multi‑stage cluster sampling with probability‑proportional‑to‑size (PPS) selection for final respondent choice [69]. An exploratory survey was used to identify cricket farmers, followed by multi-stage cluster sampling to randomly choose participants, ensuring sample sizes matched the farming clusters in each area. Surveys were administered on 20^th^ July- 6^th^ Aug 2020 and on 30^th^ Nov −12^th^ Dec 2020, in Uganda and Kenya respectively encompassing 220 cricket-farming households and 457 non-cricket-farming households within the adoption region. The cricket lifecycle is 45 days, the two periods offered an opportunity to survey in different seasons while noting that farmed crickets unlike other livestock are not adversely affected by climatic fluctuations. Trained enumerators conducted a pretest with selected respondents to identify and resolve any issues before full deployment. Data were gathered using a structured questionnaire that included topics such as farming systems, cricket farming practices, household demographics, and socio-economic factors, incorporating both open-ended and Likert scale questions related to insect farming adoption. The final section of the questionnaire addressed household food security. The questionnaire was digitized using Kobo Collect [70] to ensure consistency between mobile data entries and the original Word document.

### 3.2. Ethical consideration and consent

Ethical clearance was obtained from the Mount Kenya University Research Ethics Committee (Approval no. 765) and subsequently registered with the National Council for Science Technology and Innovations (NACOSTI) in Kenya for approval and licensing. In Uganda, ethical clearance was secured from the Research Ethics Committee at the School of Social Sciences, Makerere University (No. MAKSS REC 09.19.317) and the Uganda National Council for Science & Technology (UNCST) research no. SS 5182. In addition, the questionnaire included an introductory statement addressing confidentiality and the reporting of research findings, as well as a written request for voluntary consent to participate in the survey. All respondents were above the age of 18 years.

### 3.3. Conceptual framework

**Fig 1:**
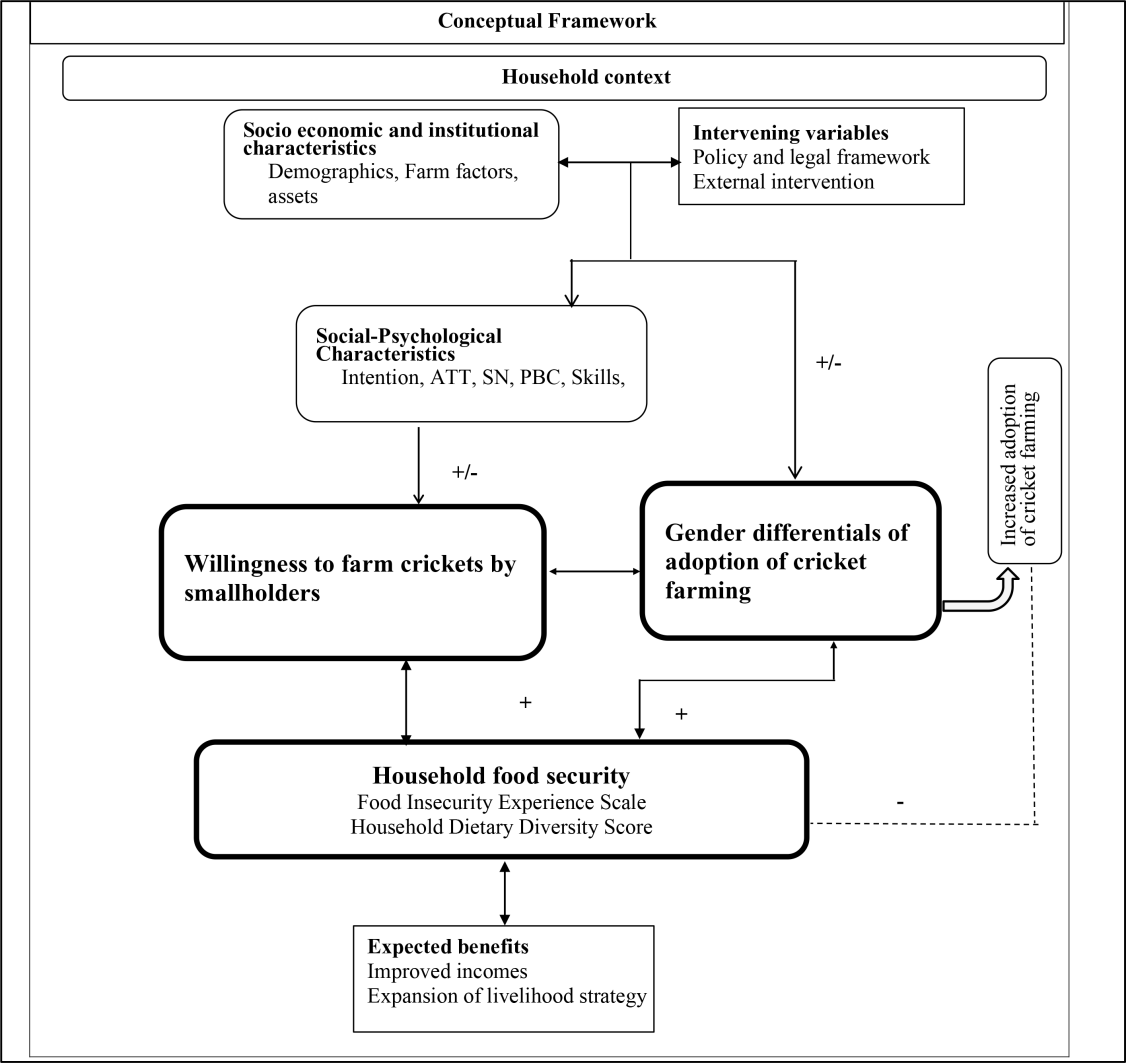
A conceptual framework of adoption cricket farming and its effect on household food security.

We present a conceptual framework in Fig 1, illustrating the relationship between the dependent and independent variables. The independent variables encompass household demographics, socio-economic factors, knowledge and perceptions of insects, and social-psychological characteristics.

Household demographics, socio-economic status, resource access, and awareness of cricket farming jointly determine the decision to adopt this innovation. The dependent variables are gendered participation in cricket farming and household food security. We hypothesized that participation in cricket farming enhances household food security, with this relationship being moderated by the demographic and socio-economic characteristics of the participants. For instance, gender is posited to play a critical role in the adoption process, as different influencing factors may be present for male (such as better access to inputs and markets) while female decision-makers may face barriers such as credit and extension services access. Larger households may provide labour for cricket production, but they may require higher food intake, offsetting the gains on increased food availability. Age and farming experience may present risk aversion or efficiency in integration of cricket farming into existing systems. Households with more assets may be better positioned to invest in cricket farming, while low-income households may adopt it as a low-cost strategy for food security. Further, higher education can raise awareness of the nutritional and economic benefits of cricket farming. It can also improve access to information and markets, further strengthening food security.

By synthesizing HUMT and RAA, the conceptual framework recognizes that the adoption of cricket farming is influenced by both rational decision-making based on economic and resource considerations and behavioral factors such as attitudes, norms, and perceptions. Participation in cricket farming is anticipated to yield improvements in food security outcomes, thereby establishing a direct link between the decision to adopt cricket farming and the resultant enhancements in food security for households.

### 3.4. Empirical model specifications and data analysis

We adopted the FAO’s Food Insecurity Experience Scale (FIES) to measure access and stability, and the Food Consumption Score (FCS) to assess utilization and availability [71–73]. Respondents were instructed to recall their consumption of a variety of foods and beverages over the preceding seven days to assess dietary diversity. Participants were to include all meals and snacks consumed by the household, regardless of whether they were prepared at home or consumed away from home. The Food Insecurity Experience Scale (FIES) was assessed through self-reported data regarding food-related behaviors within the framework of restricted access to food over the preceding twelve months, attributable to insufficient financial resources or other constraints. This assessment comprised eight questions that are integral to the FIES [72,73] which is designed to evaluate levels of food insecurity in agricultural households [4,47]. The 12‐month recall period used in the FIES can help capture seasonal and climatic influences by covering a full year of variability, which includes different seasons and any fluctuations in food security that occur over time. Based on the FIES self-assessments indices, households were classified into four categories: chronic food insecurity (persistent food shortages throughout the year), transitory food insecurity (temporary or seasonal food shortages), break-even (no food shortages, but also no surplus), and food surplus (consistent food with surplus). Chronic food insecurity is defined as the long-term or persistent inability to meet minimum dietary energy requirements while transitory food insecurity refers to a short-term (possibly temporary) inability to meet dietary energy requirements [74,75]. These classifications have been used in the methodologies and analyses of food security research studies [47,58,76,77].

Data analysis was performed using the R statistical software [78] along with the relevant packages. Participation in cricket farming was expected to improve household’s food security, the decision to participate was linked to food security outcomes by the following multiple regression equation (1);


$$\begin{array}{c} \begin{array}{c} {Fs}_{i}=\beta_{i}D_{i}+\alpha_{1}Gender+\alpha_{2}\;Age+\alpha_{3}Education+\alpha_{4}\;Marital\;status\\ \;+\alpha_{5}\;Household\;size+\;\alpha_{6}Assets+\;\alpha_{7}Land+\alpha_{8}\;Ready\;Market+\alpha_{9}MarketAcess\\ \;+\alpha_{10}\;Processing\;Tech\;Available+\alpha_{11}HGroupMembership\;+\;\alpha_{12}\;Easys\;access\;credit\\ \;+\alpha_{13}tTrain\;handling\;EI+\alpha_{14}Insect\;Inclusive\;Standards+\;\alpha_{15}Awareness\;index\\ \;+\alpha_{16}\;Perceived\;Benefits\;index+\alpha_{17}Perceived\;Risks\;index+\alpha_{18}Perceived\;Norms\;index+e_{i} \end{array} \end{array}$$ (1)

where *D*_*i*_ is the decision to adopt cricket farming, and various factors which include household characteristics, resource and market access, social economic characteristics, insect knowledge and perception which influence household food security *Fs*_*i*_, β_i_ and α_*i*_ are parameters estimated, and *e*_i_ denotes the error term.

Further, the study employed the Endogenous Switching Regression (ESR) model using the ‘endoswitch’ package in R [79], wherein the endogenous variable, i.e., decision to participate is represented as a binary (dummy) variable. The ESR model is widely utilized to address unobservable factors that influence decision-making, while also correcting for sample selection bias and heterogeneity in the data [80]. Originally developed by Lee [81] as a generalization of Heckman’s selection correction approach [82], the ESR model is frequently utilized in empirical analyses to account for self-selection bias. Although Propensity Score Matching (PSM) represents an alternative method for addressing self-selection bias, its capacity to account for biases arising from unobserved factors is limited. In contrast, the ESR model incorporated instrumental variables (IV) exerted a strong influence on the decision to participate in cricket farming while remaining exogenous to household food security- to adeptly address unobserved influences. This approach effectively addressed endogeneity concerns, enhancing the model’s robustness against omitted variable bias and serving as a validity check for the ESR. The IVs—such as Market Access, Awareness Index, and Insect-Inclusive Standards—were included to appropriately correct for the endogenous selection into cricket farming. The IV test employed Sargan overidentification test resulted in a p-value of 0.120, indicating that the IVs were uncorrelated with the error term, as the p-value exceeded the conventional threshold of 0.05. While the weak instrument test yielded a p-value of 0.001 and an F-statistic of 11.9, surpassing the critical value of 10, thus confirming the strength of the selected IVs. These diagnostic tests collectively suggested that the ESR model was properly specified and robust against omitted variable bias. Additionally, the covariate balance in matched data showed that the Kolmogorov-Smirnov (KS) test yielded a p value of 0.901, which suggested that the matching process was effective in reducing bias in the estimated treatment effect.

The estimation of the ESR model was conducted in two stages: (i) a probit regression estimates the probability of adopting cricket farming, and (ii) separate regression models estimate the determinants of food security outcomes for both cricket adopters and non-adopters. Nonetheless, this two-stage approach may result in heteroskedastic residuals, which is why the study employed Full Information Maximum Likelihood (FIML) to simultaneously estimate the selection and outcome equations [83]. Consequently, the study estimated the probability of participation in cricket farming (treatment equation *D*_*i*_) and the associated food security outcomes (outcome equation *F*_*S*_) simultaneously using full information maximum likelihood approach to account for self-selection bias. This methodology yielded two regression equations that delineate the outcomes of food security for both participants and non-participants.


$$D_{i}=1\;if\;\delta X_{i}+\varepsilon_{i}>0\;or\;D_{i}=0\;if\;\delta X_{i}+\varepsilon_{i}\leq 0$$ (2)

$$\begin{array}{c} F_{s1i}^{*}=Z_{1i}\beta_{1}+e_{1i}\;if\;Di\;=1\;(regime\;1\;–participant) \end{array}$$ (3)

$$\begin{array}{c} F_{s0i}^{*}={Z_{0i}\beta }_{0}+e_{0i}\;if\;Di=0\;(regime\;2-\;non-participant) \end{array}$$ (4)

where ${\text{F}}_{\text{s1i}}^{\text{*}}$ and $F_{s0i}^{*}$ are unobserved variable that determined the observed food security outcomes *F*_*S1*_ and *F*_*S0*_*, Xi* are vectors that determine the switch between regime 1 and 2, *Z*_*1*_ and *Z*_*0*_ are vectors of exogenous variable determining variations in the two regimes, δ, $\beta_{1}$ and $\beta_{0}$ parameters estimated and $\varepsilon_{i}$, $e_{1i}$, and $e_{0i}$ were the error terms.


$$E\left(F_{si1}^{*}|D_{i}=1;Z_{i1}\right)=Z_{i1}\beta_{1}+\sigma_{1}\lambda_{i1}$$ (5a)

$$E\left(F_{si0}^{*}|D_{i}=0;Z_{i0}\right)=Z_{i0}\beta_{0}+\sigma_{0}\lambda_{i0}$$ (5b)

$$E\left(F_{si1}^{*}|D_{i}=0;Z_{i0}\right)=Z_{i0}\beta_{1}+\sigma_{1}\lambda_{i0}$$ (5c)

$$E\left(F_{si0}^{*}|D_{i}=1;Z_{i1}\right)=Z_{i1}\beta_{0}+\sigma_{0}\lambda_{i1}$$ (5d)

Equations (5a) and (5b) are actual while (5c) and (5d) are counterfactual. Thus, the average treatment on treated (ATT) is the difference between (5a) and (5d). The average treatment effect on the untreated (ATU) is the difference between (5c) and (5b), and treatment effects are difference between ATT and ATU. The estimated counterfactual outcomes, allowed for a thorough assessment of food security differences between participating and non-participating households, independent of selection bias.

### 3.5. Description of variables

A detailed description of the explanatory variables used in the study is presented in S1 Table, which includes both continuous and categorical types. These variables are employed to explain or predict the outcomes of the response variable, aiding in the establishment of relationships and patterns in the data.

## 4. Results

### 4.1. Descriptive statistics for participation by gender

#### 4.1.1. Demographic characteristics.

The predictors in Table 1 provide an overview of gendered participation across several socio-economic and food security factors, using Chi-square analysis to reveal relationships between gender of decision maker and these variables. Approximately 67% of participants engaged in the cricket farming were females. The highest percentage of the participants in the two genders were in the age bracket of 36–50 years of age at 30.2% and 38% for male decision makers (MDMs) and female decision makers (FDMs) respectively. Regarding education, a greater percentage of females (46.3%) had completed primary education, whereas more males (44.8%) had secondary education compared to females (35.4%). Majority of the respondents were married, with a higher proportion of males (79.7%) than females (64.1%) falling into this category. The household sizes with more than 6 person (category 3 and 4), were 49% for female and 45% for the male decision makers.

**Table 1:** Gender-disaggregated descriptive statistics for demographics, resources, and market factors.

		Total (n = 644)			MDMs (n = 212)			FDMs (n = 432)		
Variable	Category	Freq	%	P-value	Freq	%	P-Value	Freq	%	P-value
***Demographic factors***										
Decision participates	0	458	71.1	<0.001	150	70.8	0.96	308	71.3	0.96
	1	186	28.9	<0.001	62	29.2	0.96	124	28.7	0.96
Age	1	62	9.6	<0.001	29	13.7	0.059	33	7.6	0.059
	2	153	23.8	<0.001	54	25.5	0.059	99	22.9	0.059
	3	231	35.9	<0.001	64	30.2	0.059	167	38.7	0.059
	4	103	16	<0.001	36	17	0.059	67	15.5	0.059
	5	95	14.8	<0.001	29	13.7	0.059	66	15.3	0.059
Education	0	15	2.3	<0.001	2	0.9	<0.001	13	3	<0.001
	1	255	39.6	<0.001	55	25.9	<0.001	200	46.3	<0.001
	2	248	38.5	<0.001	95	44.8	<0.001	153	35.4	<0.001
	3	96	14.9	<0.001	44	20.8	<0.001	52	12	<0.001
	4	30	4.7	<0.001	16	7.5	<0.001	14	3.2	<0.001
Marital status	1	77	12	<0.001	39	18.4	<0.001	38	8.8	<0.001
	2	446	69.3	<0.001	169	79.7	<0.001	277	64.1	<0.001
	3	23	3.6	<0.001	1	0.5	<0.001	22	5.1	<0.001
	4	90	14	<0.001	3	1.4	<0.001	87	20.1	<0.001
	5	8	1.2	<0.001	0	0	<0.001	8	1.9	<0.001
Household size	1	55	8.5	<0.001	28	13.2	0.031	27	6.2	0.031
	2	284	44.1	<0.001	88	41.5	0.031	196	45.4	0.031
	3	225	34.9	<0.001	70	33	0.031	155	35.9	0.031
	4	80	12.4	<0.001	26	12.3	0.031	54	12.5	0.031
***Resource and Market access factors***										
Assets	1	570	88.5	<0.001	179	84.4	0.011	391	90.5	0.011
	2	38	5.9	<0.001	13	6.1	0.011	25	5.8	0.011
	3	36	5.6	<0.001	20	9.4	0.011	16	3.7	0.011
Land	1	523	86.9	<0.001	161	83.9	0.289	362	88.3	0.289
	2	59	9.8	<0.001	24	12.5	0.289	35	8.5	0.289
	3	20	3.3	<0.001	7	3.6	0.289	13	3.2	0.289
Ready Market	1	37	5.7	<0.001	16	7.5	<0.001	21	4.9	<0.001
	2	100	15.5	<0.001	40	18.9	<0.001	60	13.9	<0.001
	3	243	37.7	<0.001	88	41.5	<0.001	155	35.9	<0.001
	4	164	25.5	<0.001	51	24.1	<0.001	113	26.2	<0.001
	5	100	15.5	<0.001	17	8	<0.001	83	19.2	<0.001
Market Access	0	373	57.9	<0.001	133	62.7	0.099	240	55.6	0.099
	1	271	42.1	<0.001	79	37.3	0.099	192	44.4	0.099
Processing Tech Available	1	47	7.3	<0.001	13	6.1	0.156	34	7.9	0.156
	2	118	18.3	<0.001	43	20.3	0.156	75	17.4	0.156
	3	265	41.1	<0.001	83	39.2	0.156	182	42.1	0.156
	4	166	25.8	<0.001	63	29.7	0.156	103	23.8	0.156
	5	48	7.5	<0.001	10	4.7	0.156	38	8.8	0.156

Significance codes p values based on Chi-square test, freq = frequency and % = percentage.

#### 4.1.2. Resource and market access factors.

Regarding assets and land, the highest percentages for both males and females were in the low category, at 84% and 83.9% for males, and 90.5% and 88.3% for females, respectively. In terms of the ready market for the crickets, most participants from both genders expressed a neutral stand. A larger percentage of males (62.7%) had no access to the market based on output marketed, as opposed to females (55.6%). About 42% of individuals from both genders had a moderate perception of access to processing technology for edible insects.

#### 4.1.3. Socioeconomic capital, insect knowledge and perception.

In Table 2, a higher percentage of males (65.1%) are not affiliated with any group compared to females (48.8%). In contrast, access to credit did not show a significant discrepancy between the genders in all categories.

**Table 2:** Gendered descriptive statistics for socioeconomic capital, insect knowledge, and perception.

		Total n = 644			MDMs n = 212			FDMs n = 432		
Variable	Category	Freq	%	P-value	Freq	%	P-value	Freq	%	P-value
***Socioeconomic capital***										
Group Membership	0	349	54.2	0.033	138	65.1	<0.001	211	48.8	<0.001
	1	295	45.8	0.033	74	34.9	<0.001	221	51.2	<0.001
Easy Access credit	1	61	9.5	<0.001	28	13.2	0.048	33	7.6	0.048
	2	139	21.6	<0.001	45	21.2	0.048	94	21.8	0.048
	3	225	34.9	<0.001	73	34.4	0.048	152	35.2	0.048
	4	162	25.2	<0.001	55	25.9	0.048	107	24.8	0.048
	5	57	8.9	<0.001	11	5.2	0.048	46	10.6	0.048
***Insect knowledge and perception***										
Train handling EI	1	26	4	<0.001	9	4.2	0.023	17	3.9	0.023
	2	102	15.8	<0.001	36	17	0.023	66	15.3	0.023
	3	239	37.1	<0.001	89	42	0.023	150	34.7	0.023
	4	184	28.6	<0.001	61	28.8	0.023	123	28.5	0.023
	5	93	14.4	<0.001	17	8	0.023	76	17.6	0.023
Insect-Inclusive Standards	1	22	3.4	<0.001	5	2.4	0.003	17	3.9	0.003
	2	71	11	<0.001	21	9.9	0.003	50	11.6	0.003
	3	368	57.1	<0.001	135	63.7	0.003	233	53.9	0.003
	4	132	20.5	<0.001	46	21.7	0.003	86	19.9	0.003
	5	51	7.9	<0.001	5	2.4	0.003	46	10.6	0.003
Awareness index	0	111	17.2	<0.001	33	15.6	0.537	78	18.1	0.537
	0.5	127	19.7	<0.001	39	18.4	0.537	88	20.4	0.537
	1	406	63	<0.001	140	66	0.537	266	61.6	0.537
Perceived benefits index	1	89	13.9	<0.001	22	10.4	<0.001	67	15.5	<0.001
	2	12	1.8	<0.001	1	0.5	<0.001	11	2.6	<0.001
	3	132	20.5	<0.001	46	21.7	<0.001	86	19.9	<0.001
	4	254	39.5	<0.001	100	47.2	<0.001	154	35.6	<0.001
	5	157	24.4	<0.001	43	20.3	<0.001	114	26.4	<0.001
Perceived risks index	1	36	5.6	<0.001	9	4.2	0.146	27	6.2	0.146
	2	180	28	<0.001	53	25	0.146	127	29.4	0.146
	3	335	52	<0.001	124	58.5	0.146	211	48.8	0.146
	4	84	13	<0.001	22	10.4	0.146	62	14.4	0.146
	5	9	1.4	<0.001	4	1.9	0.146	5	1.2	0.146
Perceived norms index	1	70	10.9	<0.001	19	9	0.24	51	11.8	0.24
	2	120	18.6	<0.001	41	19.3	0.24	79	18.3	0.24
	3	174	27	<0.001	62	29.2	0.24	112	25.9	0.24
	4	203	31.5	<0.001	72	34	0.24	131	30.3	0.24
	5	77	12	<0.001	18	8.5	0.24	59	13.7	0.24

Significance codes p values based on Chi-square test, freq = frequency and % = percentage.

Training on handling edible insects showed that a greater percentage of females, 46.1%, were in the “agree and strongly agree” category, compared to 36.8% of males. Regarding insect-inclusive standards, most respondents expressed neutrality, with a higher percentage of males (63.7%) than females (53.9%) taking this stance. Among males, 66% were aware of the use of insects as food and their farming, while 61.6% of females share this awareness. This indicates a relatively high level of knowledge among both genders, with males being slightly more informed. Most respondents expressed positive perceptions regarding the benefits of insect farming, with 67.5% of males and 62% of females indicating agreement or strong agreement. In contrast, most respondents demonstrated a neutral stance regarding perceived risk in insect farming, with 58.5% of males and 48.8% of females indicating neutrality. Additionally, only 12.3% of males and 15.6% of females expressed agreement or strong agreement with the perception of perceived risk, as reflected in the combined responses for these categories. There is minimal gender disparity in perceived norms, with 42.5% of males and 44% of females indicating agreement or strong agreement.

### 4.2. Demographic, resource, and perception factors influencing household food security by gender of decision-maker

The study explored the influence of various demographic, resource, and perception factors on household food security (HFS) across different categories (Transitory, Break Even, Surplus) in comparison to the reference category “Chronic”. Table 3 presents the outcomes of the multinomial regression analysis based on the gender of the decision maker. An odds ratio greater than 1 (OR>1) indicated that the factor increased the likelihood of achieving specific food security category relative to the reference category, an odds ratio less than 1 decreased it, and an odds ratio of 1 indicated no effect.

**Table 3:** Multinomial regression estimates of demographic, resource, and perception factors influencing household food security by gender of decision.

HFS reference category = Chronic	Household food security HFS Transitory		Household food security HFS Break-even		Household food security HFS Surplus	
*Predictors* Reference category = Male	*OR*	*std. Beta*	*OR*	*std. Beta*	*OR*	*std. Beta*
***Demographic factors***						
Gender Ã— Age	0.94	0.95	0.99	0.99	0.81	0.87
Gender Ã— Education	0.71	0.86	0.43***	0.7	0.46**	0.72
Gender Ã— Marital status	2.14**	1.37	0.94	0.97	2.13**	1.37
Gender Ã— Household size	1.40	1.13	0.97	0.98	1.16	1.04
***Resource and Market access availability***						
Gender Ã— Assets	1.00**	2.24	1.00	1.03	1.00	1.31
Gender Ã— Land	0.94	0.9	0.95	0.32	0.93	0.41
Gender Ã— Ready Market	0.44***	0.66	0.41***	0.63	0.52**	0.73
Gender Ã— Market Access	1.20	1.04	1.65	1.12	3.76***	1.36
Gender Ã— Processing Tech Available	1.04	1.01	1.73**	1.29	0.96	0.97
***Socioeconomic capital***						
Gender Ã— Group Membership	0.64	0.9	0.68	0.91	0.37***	0.8
Gender Ã— Easy Access Credit	1.26	1.14	0.86	0.93	1.04	1.03
***Insect knowledge and perception***						
Gender Ã— Train handling EI	2.02**	1.45	3.1***	1.79	2.36***	1.57
Gender Ã— Insect Inclusive Standards	1.08	1.02	2.04**	1.32	1.12	1.02
Gender Ã— Awareness index	0.43*	0.86	0.88	0.97	1.03	1.01
Gender Ã— Perceived Benefits index	1.03	1.01	1.01	1.0	1.62**	1.41
Gender Ã— Perceived Risks index	1.19	1.10	2.06**	1.34	1.06	1.05
Gender Ã— Perceived Norms index	0.96	0.98	0.74	0.85	0.7	0.82

Significance codes: ‘***’ = p < 0.001 ‘**’ = p < 0.01 ‘*’ = p < 0.05, Gender Ã: interaction terms between gender and the respective predictor.

#### 4.2.1. Demographic, resource and market access availability.

The gender interactions revealed how these factors affect male decision-makers (MDMs) and female decision-makers (FDMs) unequally in the adoption of cricket farming and the attainment of household (HH) food security. Compared to the Chronic category (reference group), older women are 19% less likely than men to attain surplus food security (OR=0.81), suggesting that age disadvantages women more than men in achieving the highest level of food security.

Education significantly reduced the likelihood of FMDs achieving Break-even and Surplus categories relative to males by 57% (OR = 0.43,) and 54% (OR 0.46) respectively, both significant at 1% level. This indicates that while education may improve food security overall, its benefits are not equitably experienced by women. Marital status appeared more beneficial for FDMs; married women were over twice as likely to attain both Transitory (OR = 2.14) and Surplus (OR = 2.13) food security levels compared to married men, both significant at the 1% level. This suggests that marital status is more advantageous for female’s food security outcomes. Household size showed positive but statistically insignificant effects for women: a 40% higher likelihood of reaching the Transitory category (OR = 1.40) and a 16% higher likelihood for Surplus (OR = 1.16). Assets ownership for FDMs showed no meaningful effect but was statistically significant for Transitory HFS (OR = 1.00, significant at the 1% level), indicating negligible change. The predictive value of land as an indicator of food security was not statistically significant across all categories.

Market-related factors reveal stark disparities. FDMs with access to a ready market were 56% less likely to achieve Transitory (OR = 0.44), 59% less likely to achieve Break-even (OR = 0.41), and 48% less likely to achieve Surplus (OR = 0.52), all statistically significant. These results highlight how limited ability to capitalize on ready market access hinders women’s food security. However, broader market access (beyond simply having a ready market) showed women with general market access were 276% more likely to reach Surplus HFS (OR = 3.76, highly significant), emphasizing the importance of market integration. Access to processing technology increased the likelihood of reaching Transitory HFS by 73% (OR = 1.73, significant at the 1% level), though its effect on Surplus (OR = 0.96) was statistically insignificant, suggesting limited impact on long-term gains without additional support.

#### 4.2.2. Socioeconomic capital, edible insect farming knowledge and perception.

Despite group membership, women remain 63% less likely to achieve Surplus HFS (OR = 0.37, highly significant), indicating persistent structural and social barriers even within collective frameworks. Access to credit has no significant effect on Break-even HFS (OR = 0.86), representing a 14% lower likelihood, while the effect on Surplus (OR = 1.04) is statistically insignificant, implying only a 4% increase. Training had a consistently strong, positive effect: FDMs were 102% more likely to reach Transitory HFS (OR = 2.02), 210% more likely to reach Break-even (OR = 3.10), and 136% more likely to reach Surplus (OR = 2.36) all significant at 1% level. These findings underscore the importance of targeted capacity-building for women. The presence of insect-inclusive standards doubled the odds of achieving Break-even (OR = 2.04, highly significant), reinforcing the role of institutional and policy frameworks.

Awareness significantly reduced the likelihood of FDMs remaining in Transitory HFS by 57% (OR = 0.43, significant at the 5% level), suggesting that well-informed FDMs were more likely to exit marginal food security. Positive perceptions of insect farming benefits are associated with a 62% greater likelihood of achieving Surplus (OR = 1.62, significant at the 1% level), while higher perceived risks increase the odds of being in the Break-even group by 106% (OR = 2.06, significant at the 1% level), suggesting hesitancy toward scaling production despite initial adoption. Lastly, perceived social norms reduced women’s chances of achieving Break-even and Surplus by 26% (OR = 0.74) and 30% (OR = 0.70), respectively, implying that societal expectations continue to pose barriers to food security gains for women.

### 4.3. Gender-specific determinants of participation in cricket farming

Table 4 presents the results of the selection model from the Endogenous Switching Regression (ESR), identifying key determinants of cricket farming adoption across gender of the decision maker.

**Table 4:** Selection model estimates for gender-specific determinants of participation in cricket farming.

	Decision to participate					
	Total n = 644		FDMs n = 432		MDMs n = 212	
Predictors	OR	Std. Beta	OR	Std. Beta	OR	Std. Beta
(Intercept)	0.03***	0.45	0.02***	0.44	0.07***	0.45
Age	0.90*	0.88	0.91	0.90	0.89	0.86
Education	1.11	1.09	1.08	1.07	1.09	1.08
Marital status	1.25**	1.22	1.24**	1.23	1.26	1.12
Household size	1.09	1.08	1.16	1.13	0.97	0.97
Assets	1.00	1.07	1.00	1.07	1.00	1.14
Land	1.00	0.88	1.00	0.85	1.02	1.10
Ready Market	0.84**	0.83	0.94	0.94	0.66**	0.65
Market Access	1.04	1.02	0.97	0.99	1.24	1.11
Processing Tech Available	1.25***	1.25	1.16*	1.16	1.53***	1.51
Group Membership	1.42***	1.19	1.48***	1.22	1.30	1.13
Easy Access credit	0.96	0.95	0.98	0.98	0.85	0.84
Train handling EI	1.11	1.12	1.02	1.02	1.52**	1.49
Insect-Inclusive Standards	0.92	0.94	0.92	0.93	0.84	0.89
Awareness index	3.08***	1.54	3.35***	1.60	2.74***	1.46
Perceived Benefits index	1.24***	1.39	1.28***	1.49	1.06	1.09
Perceived Risks index	0.78***	0.82	0.88	0.90	0.59***	0.67
Perceived Norms index	1.39***	1.47	1.32***	1.40	1.66***	1.76

Significance codes: ‘***’ =p<0.001 ‘**’= p<0.01 ‘*’ =p<0.05.

Age demonstrated a modest negative association with adoption, where each additional year reduced the likelihood of adoption by 9% (OR=0.91) for FDMs and 11% (OR=0.89) for MDMs, with an overall reduction of 10%, which is significant at the 5% level. This suggests that older individuals are generally less likely to engage in cricket farming. Education was associated with an 11% (OR=1.11) higher likelihood of adoption overall;; however, this association is not statistically significant for either gender, indicating limited direct influence of education on adoption decisions. Marital status was a significant factor for women: married FDMs are 25% more likely to adopt cricket farming (OR=1.25), a relationship that is significant at the 1% level, whereas no such effect is observed among MDMs. Similarly, although not statistically significant, household size was associated with a 16% increase in the likelihood of adoption among FDMs (OR=1.16) but showed a slight non-significant negative effect of 3% on adoption among MDMs (OR=0.97). Asset ownership and land access were not statistically significant predictors of adoption for either gender or overall, suggesting that these forms of capital did not play a decisive role in the decision to adopt cricket farming.

A ready market was associated with a 34% lower likelihood of adoption among MDMs (OR=0.66), which is significant at the 1% level, while this effect was not significant for FDMs. In contrast, market access increased the likelihood of adoption by 24% for MDMs (OR=1.24), though this effect is not statistically significant. Availability of processing technology for edible insects increased the likelihood of adoption for both genders. FDMs were 16% more likely to adopt (OR = 1.16) significantly at the 5% level, while MDMs were 53% more likely to adopt (OR = 1.53), which was highly significant, indicating that men may rely more heavily on infrastructure support. Group membership increased the likelihood of adoption by 48% for FDMs (OR = 1.48) and 42% overall (OR = 1.42) both of which are highly significant, although this effect is not statistically significant for MDMs. Easy access credit did not significantly influence adoption decisions for either gender. Training in handling edible insects significantly increased adoption for MDMs, who were 52% more likely to adopt, a result significant at the 1% level; this effect is not observed among FDMs.

Awareness played a crucial role in the adoption of both genders: FDMs who are aware of cricket farming are over three times more likely to adopt (OR=3.35)while MDMs were 174% more likely (OR=2.74). Both effects were highly significant, underscoring the importance of access to information. The perceived benefits significantly influence adoption among FDMs, who were 28% more likely to adopt (OR= 1.28). For MDMs, the effect is positive but not statistically significant(OR= 1.06). In contrast, perceived risks were a major deterrent for MDMs, reducing the likelihood of adoption by 41%, (OR = 0.59) a highly significant effect, while the corresponding 12% reduction for FDMs (OR=0.88) was not significant. Perceived norms strongly predict adoption for both genders. FDMs were 32% more likely (OR = 1.32) and MDMs 66% more likely (OR=1.66) to adopt cricket farming when normative support is present. Both results are highly significant, highlighting the powerful role of community and cultural expectations in shaping adoption behaviors.

### 4.4. Treatment and heterogeneity effects by gender of main decision maker on cricket farming

#### 4.4.1. Female decision makers.

Table 5 presents the treatment effects derived from an endogenous switching regression model for female decision makers. Female decision-makers who opted to adopt cricket farming (Y1 (adopters) demonstrated an expected food security score of 1.756, surpassing those households FDMs who chose not to adopt cricket farming (Y0 (non-adopters)), which exhibited a lower expected food security score of 1.567. The observed disparity in food security levels between FDMs adopters and non-adopters (ATT) highlights a statistically significant positive influence of cricket farming on household food security. Average Treatment Effect on the Untreated (ATU = 0.709), represents the hypothetical benefit that non-adopters would have experienced if they had adopted cricket farming among the FDMs. The heterogeneity effect of −0.520 indicates that non-adopters would have gained more from adoption than adopters did.

**Table 5:** ESR estimates of the average treatment effect for female decision makers.

	Y1 (Choose to adopt)	Y0 (Choose not to adopt)	Treatment Effect	Label
A1 (Adopted)	1.756 (0.43)	1.567 (0.29)	0.188 (0.03)	ATT
A0 (Not adopted)	2.238 (0.17)	1.529 (0.31)	0.709 (0.02)	ATU
Heterogeneity Effect	−0.483 (0.05)	0.037 (0.03)	−0.520 (0.03)	ATH

Standard deviation (in parentheses).

#### 4.4.2. Male decision makers.

The results of treatment effects for male decision makers are represented in Table 6.

**Table 6:** ESR estimates of the average treatment effect male decision makers.

	Y1 (Choose to adopt)	Y0 (Choose not to adopt)	Treatment Effect	Label
A1 (Adopted)	1.499 (0.43)	2.014(0.23)	−0.516 (0.06)	ATT
A0 (Not adopted)	2.040 (0.38)	1.673 (0.28)	0.367 (0.03)	ATU
Heterogeneity Effect	−0.542 (0.06)	0.341 (0.04)	−0.883 (0.07)	ATH

Standard deviation (in parentheses).

For MDMs cricket farmers (Y1), the average treatment effect on the treated (ATT) is estimated at −0.516 (0.06), suggesting that adoption of cricket farming may lead to a slight reduction in household food security compared to MDMs that did not adopt cricket farming (Y0). The average treatment effect on the untreated (ATU) is estimated at 0.367 (0.03), indicating a potential enhancement in food security among non-adopters if they adopted. The heterogeneity effect (ATH) is at −0.883 (0.07), showing non-adopters MDMs would have gained more from cricket farming than those adopted.

## 5. Discussion

### 5.1. Gendered characteristics of decision makers

The study showed higher female participation in cricket farming, supporting the increasing involvement of women in insect farming [84]. Most of the participants were middle aged, who are risk averse and would diversify their farming practices [85], due to economic and food security consideration. More MDMs had completed secondary education, while asset and land ownership remained low for both genders. This reflects broader social trends where males often attain higher levels of education and access to land due to historical inequalities, ultimately influencing their economic participation [86]. The larger percentages of males lacking market access and affiliation to associations indicate that male farmers face significant challenges in securing the resources and information essential from collaboration [4]. Awareness and perceived benefits of insect farming was relatively high, more so for males which shape the attitudinal aspects and utility of adoption cricket farming [84,87]. Perceptions of risk which influence perceived behavioral control were generally neutral, and perceived norms reflecting on societal expectations showed minimal gender disparity.

### 5.2. Gendered predictors effect on household food security outcomes

The gendered predictors of food security outcomes in cricket farming adoption revealed notable disparities between male decision-makers (MDMs) and female decision-makers (FDMs) (Table 3). Older females were unlikely to attaining Surplus Household Food Security (HFS) in comparison to their MDMs suggesting that age exacerbates barriers—such as limited resources and new agricultural innovations as proxy for HFS—for women [85]. While education is generally associated with improved food security, our findings indicate that its benefits are more pronounced for MDMs; FDMs faced significantly lower odds of achieving Break-even and Surplus HFS with higher educational attainment, reflecting entrenched disparities in resource access. These findings contrast with some literature that suggests education generally enhances food security [88], but may disproportionately benefit male farmers due to existing structural barriers and resource access for women [46]. Married FDMs attained higher food statuses, indicating that marriage provides additional support and resources for women. This finding aligns with research showing that marriage can improve food security by enhancing labor availability and facilitating financial resource sharing particularly for women [89]. Household size may offer additional labor resources [51,59], however, its effect on food security outcomes was not statistically significant for either gender. Suggesting that, while larger households are often linked to improved food security, in emerging economies the benefits may be offset by higher food demand, diminishing the net positive impact on food security.

Asset ownership played a critical role in shifting households from chronic to transitory food security status, demonstrating its strong buffering effect against food insecurity even in the face of climate shocks [63–65]. While land ownership does not demonstrate an impact on higher food security levels outcomes for women, this finding highlighted structural barriers that impede women’s effective utilization of land, despite their ownership underscoring the necessity for comprehensive support mechanisms for women in agriculture that extend beyond mere asset accumulation or land ownership [3,48].

FDMs with enhanced market access had elevated probabilities of achieving food security (surplus), underscoring the critical role of broader market accessibility for females. While not exclusively focused on women’s market access, Weigel et al. [66] observed that improving market access in insect farming could enhance household diets. Njuki et al. [3] further noted that limited market access and mobility further undermine women’s contributions to food security. Access to processing technology helped households transition from chronic to transitory food security, it may be more influential to adoption decision.

Group membership and access to credit did not impact food surplus level. Although group membership can enhance access to external inputs and shared knowledge, these benefits appear to be more strongly linked to the adoption of cricket farming, serving as a proxy for addressing food security challenges [88,90]. Targeted training in insect farming techniques exerts a positive effect on food security, underscoring the critical importance of capacity-building. Moreover, the adoption of insect-inclusive standards facilitated a transition from chronic to intermediate food security by legitimizing cricket products, though additional measures are necessary to drive households toward surplus production. While awareness is crucial in adoption decisions, it’s direct influence on food security was minimal. Positive perceptions and attitudes of cricket farming significantly boost the likelihood of surplus food security among women, whereas heightened perceived risks and restrictive social norms further undermine their progress. This finding aligns with research indicating that cultural norms frequently limit women’s participation in resource-intensive agricultural practices and decision-making, thereby impeding their progress toward improved food security [84].

### 5.3. Gender-specific predictors of participation in cricket farming

In line with utility maximization principles, older individuals of both genders are less inclined to adopt cricket farming, possibly due to a stronger preference for traditional practices and a lower perceived benefit-to-cost ratio, as supported by existing literature [14,91,92]. Although education is generally positively correlated with participation, its lack of statistical significance for either gender suggests that its potential benefits are muted in contexts where resource disparities persist, as supported by prior studies [93]. Marital status significantly enhances adoption among female decision-makers whereas this trend is not evident for male decision-makers. Household size positively influences FDMs’ adoption, potentially attributable to greater labor availability, but exhibits no notable impact on MDMs. This is consistent with the findings that household size is a key factor in the adoption of agricultural innovations [51].

Economic resources, including assets and land, do not emerge as significant predictors for either gender; land ownership fails to drive adoption in the context of cricket farming—a novel practice that requires minimal land compared to conventional agriculture [13]. Market dynamics further illustrate gendered differences: while a readily available market appears to deter male participation, it does not significantly affect female decision-makers; however, improved market access is more influential for males, underscoring that efficient market connectivity is critical for maximizing economic returns in a utility framework [62,91].

Technological factors, such as the availability of processing technology, exert a stronger influence on MDMs while remaining significant for FDMs. This reinforced the idea that technology adoption is integral to value addition, reducing costs and increasing returns—a core tenet of household utility maximization. Group membership played a critical role in facilitating adoption among FDMs, underscoring the importance of social networks. This is consistent with studies showing that social networks play a crucial role in promoting adoption, particularly in rural farming communities [94]. In contrast, access to credit does not significantly drive adoption for either gender, suggesting that while credit is a potential resource, it may be secondary to other factors such as collateral availability and broader resource access [88,91]. Training in handling edible insects significantly influences MDMs indicating that knowledge transfer is particularly important for male decision-makers. This concurs with research that shows training enhances confidence in adopting new agricultural practices [94]. Awareness emerges as a critical determinant for both genders. This finding supports the premise that informed decision-making—central to both utility maximization and the reasoned action approach—is vital for the sustainable adoption of cricket farming [38]. Furthermore, while perceived benefits strongly motivate FDMs to adopt, perceived risks have a greater deterring effect on MDMs, indicating that attitudes and risk aversion play a differential role in shaping the adoption process across genders [62]. Perceived norms are highly significant for both groups, underscoring that societal acceptance and the behavioral expectations of peers are pivotal in driving the decision-making process.

### 5.4. Gendered treatment effects on household food security scores

Adoption of cricket farming improved household food security for FDMs. If non-adopters had adopted cricket farming, their food security scores would have increased highlighting a substantial potential benefit. The results suggested that female decision makers are better equipped for insect farming, notably labor-intensive tasks, owing to various factors, including their nurturing skills and proficiency in overseeing household and agricultural responsibilities. Research also suggests that women commonly assume pivotal functions in small-scale farms, overseeing labor-intensive duties such as tending to livestock, feeding, and presently, insect cultivation [95]. Improvement of household food security for an adopter concurs with research conducted by Musungu et al [38] highlighting a positive correlation between participation in collective cricket farming and enhanced food security.

Male participation in cricket farming appeared to have a small negative treatment effect on household food security, with non-adopters faring better than adopters. The high initial costs and resource allocation of cricket farming can place substantial strain on household resources, particularly when managed by male decision-makers (MDMs). These start-up investments may lead to redistribution of resources from immediate consumption, potentially reducing household food availability and security in the short to medium term [96]. Although this challenge is not exclusive to male farmers, gendered patterns of intra-household resource allocation can amplify its impact. Studies indicate that men more often allocate income to non-food expenditures [97–99], implying that cricket-farming revenues controlled by male decision-makers may contribute less directly to household food security than those managed by women. Moreover, insect farming is inherently labor-intensive and demands substantial nurturing during its initial stages—an attribute more commonly associated with female capabilities than with traditional male roles [19,91]. This shift away from traditional male roles can impede MDMs’ ability to integrate cricket farming into their existing routines, potentially leading to labor inefficiencies or a diversion of time from other productive activities. These inefficiencies can diminish productivity in cricket farming and impact overall household security.

Market constraints represent a significant barrier for many MDMs. Over 62% of MDMs reported difficulties in accessing markets, reflecting underdeveloped linkages and limited involvement with marketing associations [37]. The study’s adoption model indicated that a ready market is linked to a lower likelihood of MDM participation, highlighting how perceptions of unviable markets can deter engagement. Even among those who adopt, persistent market barriers often hinder profitable sales, resulting in marginal or negative economic returns. Although collective actions, such as group membership, can alleviate labor constraints and improve market access [38], 65% of male decision-makers are not association members and therefore cannot benefit from these collaborations. As a result, these barriers prevent them from leveraging group-based strategies, which in turn undermines their ability to meet cricket farming’s labor and operational demands and to access markets. Additionally, perceived risk played a disproportionately strong deterrent role for MDMs compared to their female counterparts. While adopters may represent a more risk-tolerant subset, they still confront entrenched structural issues, such as limited buyer networks and weak institutional support [37] that hinder success. Thus, perceived risk filters entry but does not insulate male adopters from the systemic barriers that constrain outcomes.

Although the findings indicate a slightly diminished benefit for male households, the counterfactual estimates demonstrated that adoption would still yield significant improvements in food security. Addressing both structural and perceptual barriers is essential for improving male participation and ensuring sustainable food-security outcomes. Additionally, expanding access to markets and promoting collective action through farmer groups and buyer linkages are critical for mitigating the gender-specific constraints MDMs face in cricket farming.

## 6. Conclusion

The study aimed to investigate the gendered factors influencing the adoption of edible insects, such as crickets, by smallholder farmers in East Africa. It drew on the HUM and RAA frameworks to understand how utility and perception influence decisions. Endogenous Switching Regression (ESR) was used to assess the impact of choosing to adopt cricket farming on household food security outcomes for male and female decision-makers. The findings revealed a distinct gender dynamic in the adoption of cricket farming and its implications for household food security. Female decision-makers (FDMs) were more actively engaged in this practice, despite facing structural barriers and societal inequalities. Although both genders demonstrated a high level of awareness regarding insect farming, men possessed slightly more favorable perceptions, suggesting that they may anticipate higher immediate economic returns within a household utility maximization framework. While the adoption of cricket farming significantly enhanced food security for women, male decision-makers experienced lower benefits, likely due to the mismatch between expected economic returns, high labor and capital input required at the early stages of adoption. These insights underscore the necessity for targeted, gender-responsive interventions such as subsidizing start-up inputs for marginalized adopters, and formalizing cricket markets to unlock the full potential of this innovation.

## 7. Policy implications

This study identifies the role of cricket farming in enhancing nutritional security, underscoring the necessity for improved representation of cricket farmers in food policy discussions. Our findings suggest that targeted, gender-sensitive training in edible-insect husbandry significantly enhanced adoption for both genders and increased women’s likelihood of attaining higher food-security status. Consequently, training programs by research institutions (e.g., International Centre of Insect Physiology and Ecology (ICIPE), Makerere University) and NGOs should be reevaluated not only to achieve adoption success but also to enhance productivity. This will increase the quantity of crickets harvested as a proxy for improved household food security, as well as supply to processors and buyers. Because market access was found to be a key barrier, particularly for male farmer, establishing aggregation centers at the district or ward level will be an intervention at policy level to help bulk crickets and connect producers to buyers. We recommend tracking both the number of active aggregation centers and the volume of crickets sold under formal contracts. The study also found that most male decision-makers were not members of group associations. Therefore, to promote equitable access to collective resources and market opportunities, it is essential to implement targeted interventions to increase male participation in these associations. Enhanced awareness among male and female decision-makers was found to increase the likelihood of adoption. Therefore, prioritizing initiatives that raise awareness, such as radio programs and community forums, is essential. The effectiveness of these outreach efforts can be measured by tracking the number of sessions held and subsequent adoption rates disaggregated by gender.

## Supporting information

S1 Table Description of variables. (DOCX)
