# Supplementary material for: Gender differentials in cricket farming and its impact on household food security levels in East Africa
Source: PLoS One. 2025 Jun 25;20(6):e0326108. doi: 10.1371/journal.pone.0326108 (PMC12193075; doi:10.1371/journal.pone.0326108)
Supplement: S1 Table — (DOCX) [file pone.0326108.s001.docx]

S1 Table. Description of variables

| **Variables** | **Description** | **Variable type** |
| --- | --- | --- |
| **Dependent variables** |  |  |
| Decision_participate | Adoption of cricket farming (1=adopter, 0=non-adopter) | Categorical |
| Household_food_security_HFS  (For use in first regression and descriptive statistics) | Perception on food security for household (Chronic=1, Transitory=2, Break-even=3, Surplus=4) | Categorical |
| **Outcome variable (endoswitch model)** | | |
| Household_Food_Security_FIES | Food Insecurity Experience Scale household (3=Food secure, 2=Moderate food secure, 1=Severe food insecure) | Categorical |
| **Splitting variable** | | |
| Gender | Gender of decision maker (0=Male, 1-Female) | Categorical |
| **Independent variables** | |  |
| Gender | The gender of the decision maker (1=Male, 0=Female) | Categorical |
| Age | Age group of decision maker (1= 18-25 years, 2= 26-35 years, 3= 36-50 years, 4= 51-60 years, 5= Above 60 years) | Categorical |
| Education level | Highest level of education of the decision maker (0=None, 1=Primary, 2=Secondary,3=Tertiary colleges, 4=University) | Categorical |
| Marital status | Marital status of decision maker (0= Prefer not to tell,1=Single, 2=Married, 3=Separated, 4=Widowed, 5=Divorced) | Categorical |
| Household size | Number of persons in the household (1=2 and below, 2=3-5,3=6-8,4=Above 8) | Categorical |
| Employment | Employment status (0=Not employed,1=Permanent, 2=Casual,3=Self-employed) |  |
| Assets | Total household assets in USD | Continuous |
| Land | Owned land in acreage | Continuous |
| Access to market | If any output is marketed (1=Yes, 0=No) | Categorical |
| Group membership | Household member belong to a group or an association (1=Yes, 0=No) | Categorical |
| Processing tech available | Processing technologies Eis are available (1=Strongly disagree, 2=Disagree, 3= Neutral, 4=Agree, 5=Strongly agree) | Categorical |
| Ready markets | Edible insect products have a ready market (1=Strongly disagree, 2=Disagree, 3= Neutral, 4=Agree, 5=Strongly agree) | Categorical |
| Training available | Training on handling insects is available (1=Strongly disagree, 2=Disagree, 3= Neutral, 4=Agree, 5=Strongly agree) | Categorical |
| Insect inclusive standards | There are insect inclusive standards in Kenya and Uganda (1=Strongly disagree, 2=Disagree, 3= Neutral, 4=Agree, 5=Strongly agree) | Categorical |
| Access to credit | There is easy access to credit for insect farming (1=Strongly disagree, 2=Disagree, 3= Neutral, 4=Agree, 5=Strongly agree) | Categorical |
| **Awareness Index** | Median based on FEI1-FE4 | |
| Aware Insects are eaten | FEI1 Have you heard prior to survey EI are eaten (1=Yes, 0=No) | Categorical |
| Aware insects are farmed | FEI2 Are you aware that edible insects are farmed (1=Yes, 0=No) | Categorical |
| Ever consumed EI | FEI3 Have you ever consumed edible insects or EI products (1=Yes, 0=No) | Categorical |
| Farmed EI available | FEI4 Are farmed edible insect available in the village (1=Yes, 0=No) | Categorical |
| **Perceived Benefits Index** | Median based on FPB1-FPB 8 | |
| Feed conversion | FPB1 Perceived benefits farming EI Feed conversion (0=don’t Know, 1= Strongly Disagree 2= Disagree 3= Neutral 4= Agree 5= Strongly Agree) | Categorical |
| Use agricultural waste | FPB2 Perceived benefit Edible insects n agricultural waste (0=don’t Know, 1= Strongly Disagree 2= Disagree 3= Neutral 4= Agree 5= Strongly Agree) | Categorical |
| Reproduce fast | FPB3 Perceived benefit Edible insects reproduce fast (0=don’t Know, 1= Strongly Disagree 2= Disagree 3= Neutral 4= Agree 5= Strongly Agree) | Categorical |
| Less land | FPB4 Perceived benefit Less land to farm (0=don’t Know, 1= Strongly Disagree 2= Disagree 3= Neutral 4= Agree 5= Strongly Agree) | Categorical |
| Less labor | FPB6 Perceived benefit less labor intensive (0=don’t Know, 1= Strongly Disagree 2= Disagree 3= Neutral 4= Agree 5= Strongly Agree) | Categorical |
| Less environmental impact | FPB7 Perceived benefit Less environmental impact (0=don’t Know, 1= Strongly Disagree 2= Disagree 3= Neutral 4= Agree 5= Strongly Agree) | Categorical |
| World population food | FPB8 Perceived benefit the world population food production (0=don’t Know, 1= Strongly Disagree 2= Disagree 3= Neutral 4= Agree 5= Strongly Agree) | Categorical |
| **Perceived Risks Index** | Median based on perceived risks (PR1-PR4) | |
| Competition with other agricultural activities | PR1 EI increase competition to other agricultural activities (1= Strongly Disagree 2= Disagree 3= Neutral 4= Agree 5= Strongly Agree) | Categorical |
| Contamination food chain | PR2 Cause contamination of food chain (1= Strongly Disagree 2= Disagree 3= Neutral 4= Agree 5= Strongly Agree) | Categorical |
| Allergic Reactions | PR3 Cause allergic Reactions (1= Strongly Disagree 2= Disagree 3= Neutral 4= Agree 5= Strongly Agree) | Categorical |
| Negative impact to environment | PR4 Negative impact to environment if accidentally released (1= Strongly Disagree 2= Disagree 3= Neutral 4= Agree 5= Strongly Agree) | Categorical |
| **Perceived Norms Index** | Median based on SN1-SN3 | |
| People important expect I farm | SN1 People important to me expect I farm edible insects (1= Strongly Disagree 2= Disagree 3= Neutral 4= Agree 5= Strongly Agree) | Categorical |
| Most people would approve | SN2 Most people whose opinion wd approve l farm EI (1= Strongly Disagree 2= Disagree 3= Neutral 4= Agree 5= Strongly Agree) | Categorical |
| People in my social circle expect I farm | SN3 People in my social circle expect I farm EI (1= Strongly Disagree 2= Disagree 3= Neutral 4= Agree 5= Strongly Agree) | Categorical |
